# Supplementary material for: Factors related to overweight and obese populations maintaining metabolic health
Source: PeerJ. 2022 Apr 12;10:e13242. doi: 10.7717/peerj.13242 (PMC9012173; doi:10.7717/peerj.13242)
Supplement: Supplemental Information 2 [file peerj-10-13242-s002.docx]

Coding book

| Item | Variable | Code |
| --- | --- | --- |
| case_no | Case number |  |
| sex | sex | 0: women; 1: men |
| age | Age at baseline |  |
| BMI | Body mass index at baseline (kg/m^2^) |  |
| waist_bl | Waist circumference at baseline (cm) |  |
| case_p_systolic1 | Systolic blood pressure at baseline (mmHg) |  |
| case_p_diastolic1 | diastolic blood pressure at baseline (mmHg) |  |
| HTNdrug | Medication use for hypertension at baseline | 0: no; 1: yes |
| GLU | Fasting glucose at baseline (mg/dL) |  |
| GLUdrug | Medication use for hyperglycemia at baseline | 0: no; 1: yes |
| CHO | Total cholesterol at baseline (mg/dL) |  |
| TG | Triglycerides at baseline (mg/dL) |  |
| TGdrug | Medication use for hypertriglycemia at baseline | 0: no; 1: yes |
| HDL | High Density Lipoprotein-Cholesterol at baseline (mg/dL) |  |
| LDL | Low Density Lipoprotein-Cholesterol at baseline (mg/dL) |  |
| phy_act1 | Level of physical activity at baseline | 0: low; 1: moderate; 2: high. |
| Edu_gp | Level of education | 1: Illiterate/elementary school  2: High school  3: University and above |
| Marital_gp | Marital status | 1: Married;  2:single/Divorced/Separated/ Widowed/others |
| Smoke_gp | Smoking status at baseline | 1: Never smoke  2: Current smoker  3: Ex-smoker |
| Alcohol_gp | Alcohol consumption at baseline | 0: no; 1: yes |
| hyperself | History of hypertension | 0: no; 1: yes; 3: I don’t know. |
| case_cr_lip | History of hyperlipidemia | 0: no; 1: yes; 3: I don’t know. |
| diabeteself | History of diabetes | 0: no; 1: yes; 3: I don’t know. |
| case_cr_comor | History of cardiovascular disease | 1.left ventricular hypertrophy；2.coronary artery disease；3.myocardial infarction；4.congestive heart failure；5.peripheral vascular disease；6.stroke, transient ischemic attack)；7.no |
| case_physical_date | Date of follow-up | Year/month/day |
| BMI_fu | Body mass index at follow-up (kg/m^2^) |  |
| case_p_waist2 | Waist circumference at follow-up (cm) |  |
| case_p_systolic | Systolic blood pressure at follow-up (mmHg) |  |
| case_p_diastolic | diastolic blood pressure at follow-up (mmHg) |  |
| case_hyper_drug | Medication use for hypertension at follow-up | 1.Diuretics；2.ACE-Inhibitor；3.Alpha Blocker；4.Calcuium channel blockers；5.AT1-Receptor Blockers (ARBs)；6.Beta Blocker；7.None |
| Glu_lab09 | Fasting glucose at follow-up (mg/dL) |  |
| case_diabetes_drug | Medication use for hyperglycemia at follow-up | 1.Oral antidiabetic agents；2.Insulin；3.Both；4.None |
| TG_lab05 | Triglycerides at follow-up (mg/dL) |  |
| HDL_lab06 | High Density Lipoprotein-Cholesterol at follow-up (mg/dL) |  |
| case_cr_lower | Use of lipid-lowering agents | 1.Statins；2.Others；3.None |
| case_p_weight | Body weight at follow-up (kg) |  |
| case_p_height | Body height at follow-up (cm) |  |
| phy_act2 | Level of physical activity at follow-up | 0: Low; 1: Moderate; 2: High. |
| Smoke_gp2 | Smoking status at follow-up | 1: Never smoke  2: Current smoker  3: Ex-smoker |
| Alcohol_gp2 | Alcohol consumption at follow-up | 0: no; 1: yes |
