# Supplementary material for: Factors related to overweight and obese populations maintaining metabolic health
Source: PeerJ. 2022 Apr 12;10:e13242. doi: 10.7717/peerj.13242 (PMC9012173; doi:10.7717/peerj.13242)
Supplement: Supplemental Information 3 — Abbreviations: MHOO, metabolically healthy overweight/obesity; SD, standard deviation; BMI, body mass index; HDL-C, high-density lipoprotein cholesterol; LDL-C, low-density lipoprotein cholesterol. (A) Physical activity was evaluated by the International Physical Activity Questionnaire (IPAQ) Short-Form, Taiwan version. Chi-square tests were used for categorical variables. T-tests were used for continuous variables. [file peerj-10-13242-s003.docx]

**Table S1 The baseline characteristics of MHOO participants who completed follow-up and those lost to follow-up.**

|  | Participants completed follow-up | Participants lost to follow-up | *P* |
| --- | --- | --- | --- |
|  | n=89 (45.4%) | n=106 (54.4%) |  |
| Age in years, n (%) |  |  |  |
| 30-39 | 12 (13.5) | 22 (20.8) | 0.41 |
| 40-49 | 46 (51.7) | 51 (48.1) |  |
| 50-59 | 31 (34.8) | 33 (31.1) |  |
| mean age (SD) | 47.4 (5.3) | 46.1 (5.7) | 0.10 |
| Women, n (%) | 43 (48.3) | 62 (58.5) | 0.16 |
| Marital status, n (%) |  |  |  |
| Others | 7 (7.9) | 11 (10.4) | 0.55 |
| Married | 82 (92.1) | 95 (89.6) |  |
| Education, n (%) |  |  |  |
| Illiterate/elementary/senior/junior high school | 43 (48.3) | 41 (38.7) | 0.18 |
| University and above | 46 (51.7) | 65 (61.3) |  |
| Cigarette smoking, n (%) |  |  |  |
| Non-smoker | 68 (77.3) | 85 (80.2) | 0.87 |
| Smoker | 13 (14.8) | 13 (13.3) |  |
| Ex-smoker | 7 (8.0) | 8 (7.6) |  |
| Alcohol consumption, n (%) | 16 (18.0) | 17 (16.0) | 0.72 |
| Physical activity^a^, n (%) |  |  |  |
| Low | 32 (36.0) | 35 (33.0) | 0.84 |
| Moderate | 39 (43.8) | 51 (48.1) |  |
| High | 18 (20.2) | 20 (18.9) |  |
| BMI (kg/m^2^), n (%) |  |  |  |
| 24 ≤ BMI < 27 | 59 (66.3) | 77 (72.6) | 0.34 |
| BMI ≥ 27 | 30 (33.7) | 29 (27.4) |  |
| Mean BMI (SD), kg/m^2^ | 26.6 (2.5) | 26.5 (2.5) | 0.64 |
| Waist circumference (cm) | 90.0 (7.7) | 89.4 (7.2) | 0.58 |
| Systolic blood pressure (mmHg) | 130.1 (19.1) | 119.6 (14.2) | <0.001 |
| Diastolic blood pressure (mmHg) | 85.5 (12.7) | 79.7 (10.5) | <0.001 |
| Fasting glucose (mg/dl) | 92.2 (8.4) | 92.8 (8.3) | 0.60 |
| Total cholesterol (mg/dl) | 201.7 (36.3) | 201.8 (34.8) | 0.99 |
| Triglyceride (mg/dl) | 106.3 (56.8) | 101.1 (45.0) | 0.48 |
| HDL-C (mg/dl) | 56.2 (10.9) | 56.8 (11.5) | 0.68 |
| LDL-C (mg/dl) | 130.7 (30.0) | 133.2 (30.4) | 0.57 |

Abbreviations: MHOO, metabolically healthy overweight/obesity; SD, standard deviation; BMI, body mass index; HDL-C, high-density lipoprotein cholesterol; LDL-C, low-density lipoprotein cholesterol.

^a^ Physical activity was evaluated by the International Physical Activity Questionnaire (IPAQ) Short-Form, Taiwan version.

Chi-square tests were used for categorical variables.

T-tests were used for continuous variables.
